# Emerging Age-Specific Therapeutic Approaches for Dry Eye Disease

**DOI:** 10.3390/jcm14124147

**Published:** 2025-06-11

**Authors:** Tatiana Suárez-Cortés, Itxaso Herrera

**Affiliations:** 1 Research, Development and Innovation Department (R&D+I Department), FAES Farma, Av. Autonomía 10, 48940 Leioa, Spain; 2 Neuro-Ophthalmology Department and Pediatric Ophthalmology, Miranza Clínica Begoña, 48006 Bilbao, Spain; itxaso.herrera@miranza.es

**Keywords:** dry eye disease, age targeted, treatment, biomarkers, tears, ocular surface, children, young adults, elderly

## Abstract

Dry eye disease (DED) is a common, multifactorial disorder of the ocular surface. Although DED can affect individuals at any age, its prevalence, clinical manifestations, underlying mechanisms, and optimal management strategies differ considerably across the lifespan. In children, symptoms are frequently associated with atopy and allergic disorders and environmental factors, whereas in young adults, digital device usage and contact lens wear are the predominant contributors. In older adults, systemic diseases and polypharmacy significantly elevate the risk of DED. Across all age groups, tear film instability, decreased tear production, and chronic inflammation are central pathogenic features. Key tear biomarkers, such as pro-inflammatory cytokines, have been widely linked to disease development. Cathepsin S and tumor necrosis factor-alpha have recently been implicated in age-related DED. A nuanced understanding of these age-related differences is crucial for improving diagnostic accuracy and tailoring interventions to specific patient populations. This review synthesizes current evidence on DED across age groups, focusing on prevalence, risk factors, pathophysiology, molecular mechanisms, coexisting conditions, biomarkers, and treatment options. Finally, it highlights critical unmet clinical needs in the management of age-related DED.

## 1. Introduction

Since the time of Hippocrates, who first described xerophthalmia [1], our understanding of the ocular surface has evolved considerably, particularly regarding its anatomy, immunology, etiology, and the pathophysiology of dry eye disease (DED). These advancements have facilitated the development of more effective therapeutic strategies. However, it is important to recognize that the clinical management of DED continues to rely largely on subjective symptoms and observable clinical signs.

DED, as defined by the Tear Film and Ocular Surface Society (TFOS), is a multifactorial condition marked by tear film instability and the loss of homeostasis, typically accompanied by ocular symptoms. Its etiology involves tear film hyperosmolarity, inflammation, ocular surface damage, and neurosensory dysfunction [1]. These factors can initiate and sustain an inflammatory response, often leading to a self-perpetuating cycle [2]. Collectively, these manifestations fall under the classification of DED.

DED results from either reduced tear production, classified as aqueous-deficient dry eye (ADDE), or increased tear evaporation, referred to as evaporative dry eye (EDE). Mixed forms are common, often presenting with predominantly evaporative features or progressive aqueous deficiency. EDE and mixed subtypes account for over 70% of cases, while pure ADDE remains comparatively less prevalent [1,3].

In the early 2000s, DED primarily affected postmenopausal women, with studies reporting a prevalence of approximately 7.8% among women aged 50 years and older. A 2003 cross-sectional survey demonstrated an age-related increase in DED prevalence, rising from 5.7% among women under 50 to 9.8% in those aged 75 and above [4]. However, more recent data indicate a shift in the epidemiological profile of DED, revealing a broader age distribution. The condition is increasingly diagnosed in younger individuals of both sexes, a trend largely attributed to the widespread use of smartphones and computers [5]. Prolonged use of handheld electronic devices is now recognized as a significant risk factor for DED, primarily due to reduced blink rates and increased tear film evaporation [6]. This growing reliance on digital screens may contribute substantially to the rising incidence of DED.

Notably, the clinical manifestations and underlying mechanisms of DED vary across the lifespan, with distinct patterns observed in children, young adults, and the elderly [7,8,9,10]. Now recognized as a major public health concern, DED affects millions worldwide, imposing considerable economic costs and negatively impacting quality of life (QoL) [11,12,13]. The increasing prevalence among pediatric and young adult populations [14] underscores the need for heightened scientific and clinical attention.

Despite significant research progress, the diagnosis of DED continues to rely predominantly on subjective symptom reporting and observable clinical signs. There is an urgent need for validated, objective biomarkers to improve diagnostic precision, enable more accurate disease classification, and support the development of age-appropriate, targeted therapeutic strategies. While clinical evaluation remains essential, symptom perception varies markedly with age.

Emerging insights into the inflammatory and pathological changes in ocular surface tissues, particularly the conjunctiva and cornea, highlight age-related differences in disease pathways. It is increasingly evident that the triggers, progression, and consequences of DED are not uniform across age groups. In pediatric cases, the potential risk of amblyopia necessitates prompt, effective, and minimally invasive treatment, as well as proactive measures to prevent exacerbation.

In light of these considerations, this review advocates for an age-stratified classification of DED, recognizing that a nuanced understanding of age-related variations is critical to enhancing diagnostic accuracy, tailoring patient-centered interventions, and ultimately improving clinical outcomes and QoL across the lifespan. To the best of our knowledge, this is the first study to propose considering an age-based classification of DED and to align corresponding management strategies with the distinct needs of each age group at the time of disease onset. The objective of this review is to analyze the differences in DED presentation among children, young adults, adults, and the elderly based on the latest evidence. In doing so, we aim to identify unmet clinical needs and promote the development of diagnostic and therapeutic strategies that are appropriately adapted to each age group.

## 2. Methods

A non-systematic narrative review was conducted through a literature search in PubMed, covering publications from January 2010 to the time of writing this manuscript. The search was designed to capture a broad range of studies addressing epidemiology, pathophysiology, diagnostic approaches, biomarkers, and treatment strategies of DED across different age groups. The search strategy included individual and combined keywords, such as “dry eye disease” OR “DED”, AND “pediatric” OR “young” OR “childhood”, AND “age-related” OR “elderly”, AND “diagnosis”, “treatment”, “risk factors”, “management”, “prevalence”, “biomarkers”, “ocular surface”, “inflammation”, and “tears”. Studies involving all populations were considered. Only articles published in English were included.

**Figure 1:**
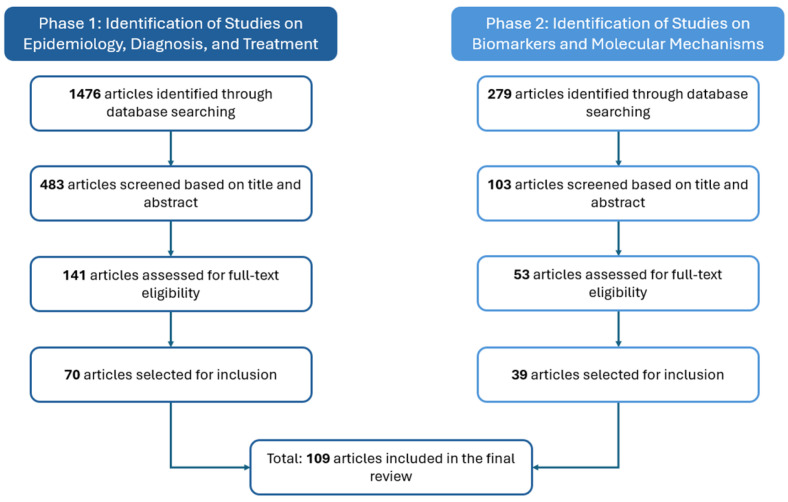
Flowchart of the study selection process.

The article selection process was conducted in two phases (Figure 1). In the first phase, we identified studies addressing the epidemiology, diagnosis, treatment, and risk factors of DED using a combination of search terms: “dry eye disease”, “pediatric”, “young”, “childhood”, “age-related”, and “elderly”. This initial search yielded 1476 articles. Filtering for studies that specifically included the terms “diagnosis” and “treatment” reduced this number to 483. We excluded studies involving animal models and those not directly related to DED, resulting in 141 articles for full-text review.

In the second phase, we performed an independent search focused on identifying key biomarkers and molecular mechanisms associated with DED. This search targeted high-impact publications using the terms “dry eye disease”, “biomarkers”, “tears”, and “ocular surface”, yielding 279 articles. From these, we selected studies that also addressed inflammation. Titles and abstracts were screened to identify articles describing the expression, correlation, and diagnostic relevance of biomarkers. Studies involving animal models or not specifically addressing DED biomarkers were excluded. A total of 53 articles met the inclusion criteria and were reviewed in full.

Since only pediatric DED is commonly recognized and used in the literature, this study introduces an age-based categorization as follows: PeDED for individuals under 18 years of age; young adult DED (y-aDED) for those aged 18 to 40 years; adult DED (aDED) for individuals between 40 and 60 years; and elderly DED (eDED) for those over 60 years of age.

## 3. Epidemiology

**Figure 2:**
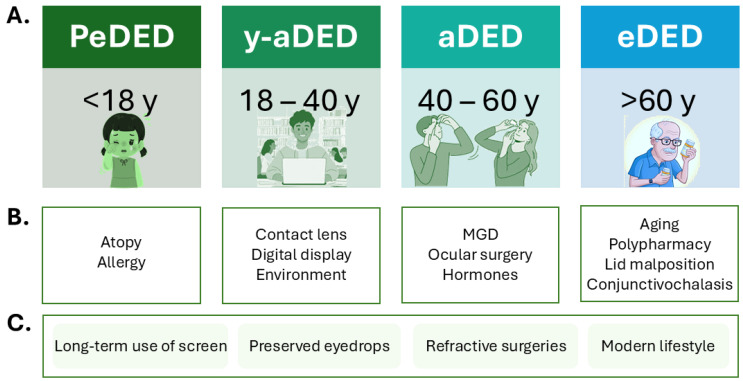
Age-based classification of dry eye disease (DED) and associated risk factors. (**A**) Classification of DED according to age group: pediatric DED (PeDED), <18 years; young adult DED (y-aDED), 18–40 years; adult DED (aDED), 40–60 years; elderly DED (eDED), >60 years. (**B**) Major risk factors currently associated with each age group. (**C**) Risk factors common across all age groups.

The prevalence of DED varies widely depending on the population studied, diagnostic criteria, geographic region, and participants’ age [11,15,16,17]. However, no standardized age-based classification of DED currently exists. Studies often differ in how they stratify age groups: some define “young” as <40 years and “elderly” as >60 years [10], while others employ more detailed categories such as young adults (19–40 years), middle-aged adults (41–60 years), and older adults (61–93 years) [18]. Pediatric studies are also heterogeneous, with some grouping all individuals under 18 as a single cohort, and others following more nuanced classifications, such as those provided by the American Academy of Pediatrics (AAP): infancy (<1 year), toddlerhood (1–2 years), early childhood (3–5 years), middle childhood (6–11 years), early adolescence (12–18 years), and late adolescence (19–21 years) [19]. A proposed classification of DED groups based on age is presented in Figure 2.

Estimating the prevalence of DED in children is particularly challenging due to heterogeneous diagnostic criteria and the frequent under-reporting of symptoms [9,14]. Additionally, evidence remains limited in this age group. A recent systematic review and meta-analysis [9] estimated a global DED prevalence of 23.7% among children (95% CI: 18.5–28.9%) (Table 1). Prevalence was notably higher when based solely on self-reported symptoms (34.6%) compared to objective clinical signs (16.6%). Moreover, the prevalence increased significantly after the COVID-19 pandemic (44.1% vs. 18.7% pre-pandemic), likely due to increased screen exposure. Climatic and environmental factors, such as latitude and average temperature, also appear to influence prevalence, along with rising rates of atopic conditions like allergies and dermatitis [20].

A large cross-sectional study [19] from India involving nearly 260,000 individuals under 21 years of age identified 1023 DED cases (0.4%). Age-specific analysis, based on the AAP classification, showed the following distribution among DED cases: infancy (1.4%), toddlerhood (2.0%), early childhood (4.8%), middle childhood (16.3%), early adolescence (38.5%), and late adolescence (37.1%). Excluding the late adolescent group, children represented 62.9% of the DED population in this cohort. The overall prevalence across the entire study population is detailed in Table 1.

Data on y-aDED prevalence (18–40 years) are less abundant than in older populations, despite this group being increasingly exposed to risk factors such as prolonged screen use and contact lens wear [10,11,31,32]. In the same Indian study, the late adolescent group (19–21 years) accounted for 37.15% of all DED cases, corresponding to a prevalence of 0.15% in the total population (Table 1) [19]. Studies on visual display terminal (VDT) users, a population that largely overlaps with young adults, report high DED prevalence rates. One meta-analysis including over 11,000 VDT workers across 16 studies estimated a global prevalence of 49.5% (95% CI: 47.5–50.6%), with individual study estimates ranging from 9.5% to 87.5% [21]. Another review of 57 studies reported prevalence rates between 26% and 70%, with VDT use strongly associated with DED signs and symptoms [22]. However, most of these studies lacked detailed age reporting. In the largest of these, conducted in Japan, the participants ranged from 22 to 60 years old, with higher prevalence observed among women, contact lens users, and individuals with prolonged screen exposure [23].

Additionally, a global estimation suggests that 10–20% of individuals over 40 report moderate-to-severe DED symptoms or seek treatment [11].

Concerning age, a large systematic review and meta-analysis [15] reported a DED prevalence of 9.2% in individuals aged 60 years and older. However, variation in diagnostic criteria across studies may impact these figures. Another study found that individuals over 40 were more likely to report moderate-to-severe symptoms, with an odds ratio of 1.3 (95% CI: 1.1–1.6) compared to younger adults [10]. In very old Russian populations, the prevalence may be even higher, since one study reported a rate of 35.8% among participants with a mean age of 84.5 years [25].

Geographic differences also play a role. In the United States, a meta-analysis [16] reported a DED prevalence of 8.1% (95% CI: 4.9–13.1%) and a meibomian gland dysfunction (MGD) prevalence of 21.2% (95% CI, 7.2–48.3%) across nearly 10 million individuals. Incidence data showed 3.5% in adults and 7.8% in those aged 68 and older.

In Spain, a cross-sectional survey conducted in 2022 estimated a DED prevalence of 16.6% based on the Women’s Health Study (WHS) criteria and at 22.5% using the Beijing Eye Study (BES) criteria. Risk factors included prior ocular surgery and the use of antidepressants, antihypertensives, and sleep aids (*p* < 0.0001), as well as digital screen use (>6 h/day). Notably, young adults (18–29 years) reported a high symptom burden but low rates of clinical diagnosis, suggesting the need for increased awareness and screening in this group [24]. Another study from a DED specialty clinic in Norway highlighted sex and age differences in DED presentations [13]. One meta-analysis estimated a pooled DED prevalence in any population in Asia of 20.1% (95% confidence interval (CI): 13.9–28.3%). The highest prevalence was 28.9% (95% CI: 6.8–51.1%) in persons ≥70 years of age, and the lowest was 7.5% (95% CI: 6.1–8.9%) in young adults (20–29 years) [26] (Table 1). Another reported a range from 5% to 50%, depending on the country and study population [33]. In China, a systematic review found an overall prevalence of DED by symptoms and signs of 13.55% (95% CI = 10.00–18.05) and by symptoms of 31.40% (95% CI = 23.02–41.13) in Chinese people aged 5–89 years [27]. In Latin America, a meta-analysis reported national DED prevalence rates of 13% (95% CI, 12–14%) in Brazil and 41% (95% CI, 39–44%) in Mexico. The meta-analyses also suggested that DE prevalence was 70% among indoor workers (95% CI, 56–80%), 71% among students (95% CI, 65–77%), and 83% in general ophthalmology clinics (95% CI, 77–88%) [28]. In the same study, MGD prevalence ranged from 23% among indoor workers (95% CI, 16–31%) to 68% in general ophthalmology clinics (95% CI, 62–72%). MGD prevalence ranged from 23% to 68%, depending on the setting. However, age-specific analyses were limited.

In Brazil, de Castro [29] reported age-stratified prevalence as follows: 18–39 years (9.9%), 40–60 (13.2%), and 60+ (21.1%). In Mexico, data using the DEQ-5 questionnaire indicated DED prevalence of 36% in those aged 46–55, 18% in those aged 66–75, and 38% in those aged 76–85 [30].

In summary, the prevalence of DED increases significantly with age and is particularly high in elderly populations. Nevertheless, DED is also prevalent in children and young adults, with contributing factors such as increased screen time, environmental exposures, and the post-pandemic digital lifestyle shift playing key roles.

## 4. Symptoms

The presentation of DED symptoms varies significantly across age groups, influenced by factors such as symptom articulation, pain perception, and comorbid ocular surface conditions [14,19,34].

In pediatric patients, clinical assessment is particularly challenging, as children often struggle to verbalize typical DED symptoms such as dryness, burning, or a gritty sensation [34]. Instead, manifestations may include excessive tearing, often a reflex response to dryness, frequent eye rubbing, a sensation of a foreign body, photophobia, contact lens intolerance, ocular redness, and difficulty maintaining focus during visual tasks like reading or schoolwork [19]. Parents may report signs such as frequent blinking or heightened sensitivity to wind or smoke. Notably, despite the presence of clinical signs, children with DED tend to report milder subjective symptoms compared to adults [14]. Clinical history in this age group should focus on personal and family histories of atopy or allergies, habitual eye rubbing, seasonal patterns of ocular redness, and visual behavior. Regular assessment of visual acuity is essential at each visit, given the potential long-term impact of ocular surface disorders on visual development, including amblyopia.

**Figure 3:**
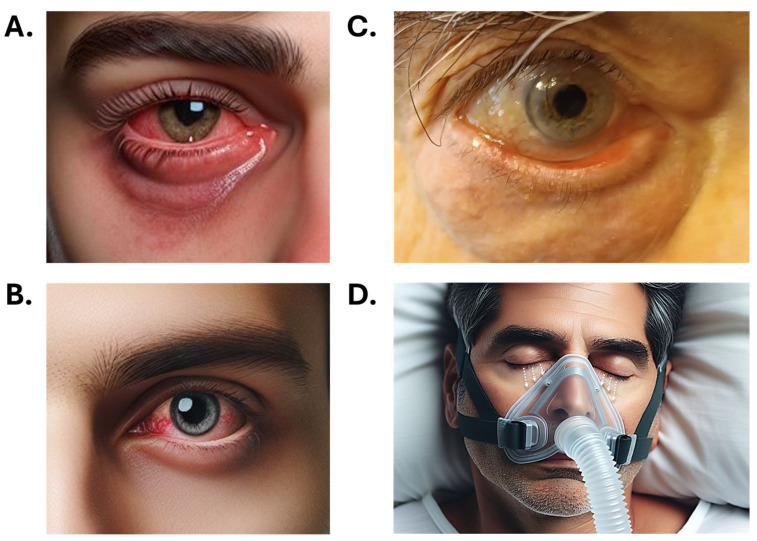
Representative images of DED across different age groups. (**A**) Young male with DED associated with atopy. (**B**) Young adult with DED related to excessive digital screen use. (**C**) Elderly male (92 years old) with DED secondary to ectropion. (**D**) Elderly male using a CPAP mask for sleep apnea; white arrows indicate mechanical traction on the lower eyelids caused by the mask. Images (**A**,**B**,**D**) were generated using artificial intelligence.

Among young and middle-aged adults, symptoms often correlate with lifestyle risk factors such as prolonged digital device use, which leads to decreased blink rates. This group commonly reports classic DED symptoms, including ocular dryness, burning, stinging, grittiness, foreign body sensation, and fluctuating vision that often improves with blinking (Figure 3). Additional complaints may include photophobia, eye fatigue, and discomfort with contact lens use, particularly after extended visual tasks [11,12,35].

Perimenopausal and postmenopausal women may experience an exacerbation of DED symptoms, highlighting the potential role of hormonal changes in disease pathophysiology. However, symptom perception can vary widely between individuals and does not always align with objective clinical findings. In elderly patients, the relationship between DED symptoms and MGD has been a focus of recent investigation, aiming to better understand the unique impact of glandular dysfunction in this population.

Older adults frequently report symptoms such as dryness, burning, grittiness, foreign body sensation, pruritus, and blurred vision that often improve with blinking [36]. Additional complaints may include eye fatigue and difficulty performing prolonged visual tasks. A notable feature in this group is paradoxical tearing, often resulting from ocular surface irritation secondary to lid malpositions (e.g., ectropion and entropion), eyelid laxity, or conjunctivochalasis (Figure 3). These anatomical changes can disrupt tear film homeostasis and trigger reflex tearing of poor quality. Despite significant ocular surface pathology, reduced corneal sensitivity in older adults may lead to the under-reporting of symptoms [37,38,39]. Furthermore, DED-related visual disturbances may be more pronounced in this population and are often misattributed to comorbid age-related conditions such as cataracts or glaucoma.

## 5. Risk Factors

DED arises from a complex interplay of demographic, systemic, ocular, iatrogenic, and lifestyle-related factors, whose impact and prevalence vary across age groups [11] (Figure 2). Risk factors can be classified as modifiable, such as screen time, stress, or environmental exposure, and non-modifiable, including age and biological sex.

### 5.1. Pediatric Population

In children, non-modifiable risk factors are often linked to underlying ocular or systemic diseases. A recent expert consensus [40] highlights environmental and lifestyle factors, particularly excessive screen time and ocular allergies, as key contributors to PeDED. A comprehensive narrative review [14] further reports higher DED prevalence in children with ocular allergies and systemic conditions. Ocular allergies can disrupt tear film stability and induce ocular surface inflammation, making them a recognized comorbidity in PeDED [41,42,43,44].

MGD is another notable cause of evaporative DED in children. An Indian cross-sectional study found MGD to be the leading cause of EDE in adolescents, while aqueous-deficient DED (ADDE) was more common in infancy and early childhood, often secondary to conditions like Stevens–Johnson syndrome (SJS) and vitamin A deficiency (VAD) [19].

Anterior blepharitis, particularly the scaly (seborrheic) form, is more prevalent in childhood than posterior blepharitis and may involve secondary staphylococcal infections, contributing to ocular surface inflammation [45].

Atopic dermatitis (AD) is frequently associated with ocular surface disease (OSD), including DED. In a clinical study assessing the ophthalmological impact of AD, OSDs were identified in 85% of patients with AD, with DED being the most prevalent (64%), followed by allergic conjunctivitis (AC) (42%), posterior blepharitis (33%), and anterior blepharitis (27%). Notably, OSDs were also detected in 63% of AD patients without ocular symptoms, although these cases were predominantly mild [46].

Although less common, DED is also associated with autoimmune and inflammatory disorders, such as juvenile idiopathic arthritis (JIA), Sjögren’s syndrome (SS), graft-versus-host disease (GVHD), and SJS [14]. Additionally, systemic conditions, including diabetes, obesity, insulin resistance [47], and VAD, have been implicated in PeDED, often leading to ADDE [14].

Among modifiable risk factors, prolonged digital screen use is increasingly recognized, as it leads to reduced blink rate and increased tear evaporation [9,11,48]. Also, exposure to dry environments and tobacco smoke further exacerbates tear film instability, contributing to PeDED.

### 5.2. Risk Factors in Young Adults

In young people and adults, the etiology of DED becomes more complex. The female sex is also identified as a predisposing factor in this age group [13]. MGD continues to be a leading cause of EDE, particularly in late adolescence (19–21 years), potentially associated with seborrheic dermatitis and acne [19]. Also, refractive procedures such as LASIK, PRK, LASEK, and SMILE can induce transient or chronic DED in some adults [49]. Therefore, preoperative evaluation for any ocular surgery should include a thorough assessment of the tear film to minimize the risk of severe postoperative DED. Surgical and pharmacological iatrogenesis may unmask pre-existing, asymptomatic DED. When indicated, appropriate preventive measures should be implemented, including patient education and the initiation of prophylactic treatment.

Digital screen use remains a predominant modifiable risk factor, leading to decreased blink frequency, incomplete eyelid closure, and increased MGD [11,23,31]. During the COVID-19 pandemic, increased screen time was associated with a significant rise in the prevalence of DED, particularly among females and individuals with more than four hours of daily visual display use [50]. Based on the Ocular Surface Disease Index (OSDI) questionnaire, the prevalence of symptomatic DED among Chinese high school students during the COVID-19 outbreak was 70.5% [50], and it was 51.8% among Spanish university students [51]. Among Indian medical students, pre-pandemic prevalence reached 46.1% [52], with longer screen exposure (>6 h/day) strongly associated with symptomatic DED.

Contact lens wear is another major risk factor, contributing to tear film instability and inflammation [11]. Environmental exposures such as air conditioning, pollution, and low humidity also exacerbate symptoms and are considered modifiable lifestyle factors [53]. Concurrent exposure to multiple risk factors (e.g., screen use, contact lenses, and environmental triggers) significantly increases the risk of ocular surface damage.

Cosmetic use and poor eyelid hygiene may further disrupt the tear film and meibomian gland function [54], adding to the modifiable behavioral risks [55].

### 5.3. Risk Factors in Elderly

In older adults, age is the principal non-modifiable risk factor associated with degenerative changes in the lacrimal and meibomian glands [8,10,15,38]. The female sex also remains a significant predisposition factor, likely due to hormonal changes, with elderly women showing higher DED prevalence [11,13].

Polypharmacy is common in this group and represents a major iatrogenic risk factor. Medications such as antidepressants, antihistamines, hormone replacement therapy, and topical glaucoma treatments, especially those containing preservatives like benzalkonium chloride, are known contributors to DED [11,56,57]. Age-related anatomical and functional changes in the eyelids, such as lid laxity, orbicularis muscle weakening, and canthal tendon stretching, further impair tear distribution [8,58,59]. Conditions like ectropion, entropion, lagophthalmos, floppy eyelid syndrome, and blepharospasm hinder proper corneal protection and tear film stability, reducing tear break-up time and leading to DED (Figure 3C). Malpositioned eyelids have been associated with DED in up to 70% of cases [60]. Additionally, sleep apnea and the use of continuous positive airway pressure (CPAP) devices for treatment are linked to eyelid laxity and nocturnal exposure keratopathy [36,61,62] (Figure 3D). Previous ocular surgeries, such as cataract extraction, may further destabilize the ocular surface and exacerbate symptoms [63].

In summary, across all age groups, DED is a multifactorial disease influenced by an intricate combination of non-modifiable factors (e.g., age, sex, and systemic conditions) and modifiable elements (e.g., digital screen time, environmental exposure, medication use, and lifestyle behaviors). Effective prevention and management require a tailored approach that accounts for age-specific risk profiles and modifiable triggers.

## 6. Diagnosis

The diagnosis of DED requires a comprehensive approach, combining symptom assessment, the evaluation of tear film quality and quantity, and examination of the ocular surface [64]. However, diagnostic strategies must be adapted to the specific needs of each age group.

In children, diagnosis relies heavily on meticulous history taking from both the child and their parents, as well as thorough clinical examination. Although adult-oriented questionnaires may be employed, their validity in pediatric populations is limited [5,9]. Clinical history must be exhaustive, often requiring repetitive questioning to uncover predisposing factors for ocular symptoms. Follow-up observations by parents can provide valuable information. Key areas of inquiry include the presence of allergies, dermatological conditions, seasonal patterns of ocular redness, photophobia, frequent eye rubbing, comments from teachers, and changes in ocular symptoms during vacations.

Clinical evaluation includes the tear break-up time (TBUT), Schirmer’s test (with or without anesthesia), although it may not always be feasible in young children, and ocular surface staining with fluorescein and lissamine green. Examination of the inferior and, if possible, superior tarsal conjunctiva is crucial to detect papillary reactions, mucous secretion, or signs of inflammation. Meibomian gland assessment is particularly important. Point-of-care tests, such as tear osmolarity, may offer additional diagnostic value; however, they are not routinely available in all settings and require further validation in pediatric populations [19,65].

In y-aDEDs, diagnostic evaluation typically involves symptom assessment using validated questionnaires, such as the OSDI or the Standardized Patient Evaluation of Eye Dryness (SPEED) [66]. Objective clinical tests may include the measurement of tear osmolarity, assessment of tear film stability (TBUT or non-invasive TBUT), evaluation of the tear volume through Schirmer’s test or tear meniscus height, and ocular surface staining [64]. Additionally, MGD, including both gland expressibility and secretion quality, should be thoroughly evaluated, particularly in contact lens users or individuals with high levels of digital screen exposure.

In elderly patients, diagnostic principles are similar to those applied in younger adults but require the consideration of specific age-related factors. Reduced corneal sensitivity and the presence of coexisting ocular conditions may result in the under-reporting of symptoms [38]. Therefore, clinical signs may precede subjective complaints. An assessment of blink dynamics and eyelid laxity is particularly pertinent in this group. Also, tear osmolarity serves as a useful objective marker. Furthermore, the impact of systemic medications on tear production and ocular surface health must be carefully evaluated [11,13,56]. In many cases, family members may report changes in ocular behavior in patients with cognitive decline or neurological disorders, such as advanced Parkinson’s disease, where the patient may be unable to communicate their symptoms effectively.

## 7. Pathophysiology

The underlying pathophysiological mechanisms of DED are complex and multifactorial, involving interactions among deficient tear secretion, increased tear evaporation, ocular surface inflammation, and neurosensory abnormalities [67]. These mechanisms can vary across different age groups [12,13,67].

In children, pathophysiology often reflects the primary underlying etiology. In cases of staphylococcal blepharitis, contamination contributes to tear hyperosmolarity and ocular surface inflammation [19]. Allergy-driven inflammation is another major factor, with AC significantly impacting ocular surface health in this population. In uncommon conditions such as SJS and VAD, the primary issue lies in aqueous tear deficiency and damage to the conjunctival epithelium, including goblet cell loss, leading to tear film instability [19,48,49]. Similar to adults, both aqueous deficiency and increased evaporation may contribute to pediatric DED, with environmental exposures and digital screen use serving as important exacerbating factors [31]. In some children, MGD may be related to incomplete or abnormal gland development, further promoting EDE.

y-aDED is frequently driven by evaporative mechanisms, primarily due to MGD. Prolonged digital screen use is a significant contributor, reducing the blink rate and enhancing tear film evaporation [12]. A study comparing tear film lipid layer thickness (LLT) in y-aDED and eDED patients found that younger individuals exhibited more symptoms and a higher blink frequency, likely related to a smaller LLT, as the meibomian gland structure and function were less deteriorated than in older patients [68].

Additionally, contact lens wear is common in this age group and can disrupt the tear film, exacerbating evaporation. Ocular surface inflammation, induced by tear hyperosmolarity and mechanical stress, plays a central role [67]. Neurosensory abnormalities may further contribute to symptom severity [10,69].

In the elderly, numerous age-related changes predispose to DED [15,38]. These include decreased lacrimal gland function, aqueous tear production [1,59], and MGD with altered lipid secretion [70]. Aging also promotes ocular surface inflammation through immune dysregulation (“inflammaging”) [15,71], goblet cell loss with consequent mucin deficiency [72], and diminished corneal sensitivity, impacting blink reflex and tear secretion. Oxidative stress increasingly contributes to ocular surface damage with advancing age [73,74]. Specifically, lacrimal gland dysfunction with aging involves senescence of the acinar cells, reduced innervation, and impaired secretory responses, often being accompanied by decreased gland volume. Meibomian glands similarly undergo ductal dilatation, acinar atrophy, and compositional changes in lipid secretion [59,70,75]. Chronic ocular surface inflammation, amplified by age-related increases in inflammatory mediators such as tumor necrosis factor-alpha (TNF-α) [15,71], is a hallmark of DED in the elderly. Furthermore, reduced corneal sensitivity with age diminishes the blink rate and tear film stability.

Overall, inflammation, neurosensory abnormalities, and oxidative stress are central to DED pathophysiology across all age groups, although the relative contribution of each factor varies with age.

## 8. Coexisting Diseases

The prevalence of systemic and ocular diseases associated with DED varies significantly across age groups. The recognition of comorbidities is essential at all ages, as they can influence both the clinical presentation and therapeutic response of DED.

In children, systemic conditions, such as SJS, VAD, and allergic diseases, are prominent causes of DED [19,36]. AD can affect both the eyelids and ocular surface, contributing to dry eye symptoms. Ocular and systemic allergies are frequently encountered in pediatric patients and can coexist with or exacerbate DED [76] (Table 2).

In young adults and adults, DED may coexist with mental health disorders, including anxiety, depression, migraine, and sleep disturbances [77]. The widespread use of contact lenses in this group also predisposes contact lens-related dry eye. Autoimmune diseases, although less frequent in young adults compared to older populations, can manifest with ocular involvement; thyroid dysfunction, notably Graves’ disease and Hashimoto’s thyroiditis, have been associated with DED (Table 2). Mechanisms include autoimmune attacks on orbital tissues, hormonal imbalances affecting tear production, and systemic inflammation impacting the ocular surface. Alterations of the tear film in patients with thyroid disease have been documented [78].

In the elderly, systemic autoimmune diseases, such as SS and rheumatoid arthritis, are more prevalent and strongly associated with DED [36]. SJS is a key contributor to both ocular and oral dryness. Diabetes mellitus, common in this age group, increases DED risk and may impair corneal sensitivity and lacrimal gland function. Ocular comorbidities, including glaucoma, particularly in patients treated chronically with preservative-containing eye drops [57,71], and age-related macular degeneration (AMD), may further complicate DED management. Furthermore, systemic lupus erythematosus and rheumatoid arthritis are increasingly common among the elderly and frequently associated with dry eye symptoms. Xerostomia, another manifestation of mucosal dryness in older adults, is often found alongside DED (Table 2) [36,79]. Thus, a comprehensive evaluation of systemic and ocular comorbidities is crucial for the appropriate diagnosis and management of DED across all age groups.

## 9. Molecular Mechanisms and Biomarkers

At the molecular level, DED is characterized by the dysregulation of multiple pathways and mediators that critically influence its onset and progression. Inflammation driven by cytokines plays a central role [80].

Inflammation, hyperosmolarity, and oxidative stress constitute the core pathological mechanisms underlying DED. Hyperosmolarity plays a pivotal role by directly damaging ocular surface tissues or indirectly triggering inflammation. This initiates a cascade of intracellular signaling events in the epithelial cells of the ocular surface, resulting in the release of pro-inflammatory mediators. These processes contribute to the self-perpetuating vicious cycle characteristic of DED, which involves tear film hyperosmolarity, inflammation, and damage or loss of epithelial and goblet cells. Such damage leads to decreased ocular surface wettability and tear film instability, manifested as early tear film breakup, thereby exacerbating hyperosmolarity and perpetuating disease progression.

Inflammation also facilitates the recruitment of immune cells, which serve as additional sources of inflammatory mediators. At the molecular level, these cascades include the activation of the JNK signaling pathway, promoting the expression of matrix metalloproteinase-9 (MMP-9) in human corneal epithelial cells. Key inflammatory mediators implicated in DED pathogenesis include interleukin-1β (IL-1β), tumor necrosis factor-alpha (TNF-α), interferon-gamma (IFN-γ), and interleukin-17 (IL-17), all of which contribute to corneal barrier disruption. Moreover, chemokine receptors such as CCR6 and CXCR3 are essential for CD4^+^ T-cell-mediated responses, while the CCR6/CCL20 axis specifically mediates Th17 cell migration. These inter-related cellular and molecular pathways collectively drive the pathogenesis of DED [67].

While the molecular mechanisms underlying pediatric DED are less extensively explored, in allergy-related childhood DED, elevated levels of histamine and various inflammatory cytokines have been detected in tears. Similarly, in young adults, inflammatory cytokines such as IL-1β and TNF-α, along with matrix metalloproteinases (MMPs), are implicated in DED pathogenesis, particularly in association with contact lens wear and ocular surface stress related to digital device use [12]. Environmental dryness, allergens, and contact lens use can provoke the release of inflammatory mediators and chemokines into the ocular surface and tear film. Among these, MMP-9 is notably elevated in the tears of DED patients, contributing to the disruption of the corneal epithelial barrier.

Matrix metalloproteinase-9 (MMP-9, also known as gelatinase B) is an enzyme involved in the degradation of extracellular matrix components, particularly collagen types IV and V. It is produced by the corneal epithelium, infiltrating leukocytes, fibroblasts, and lacrimal glands, and is secreted as an inactive zymogen (proMMP-9). This precursor undergoes proteolytic activation by various enzymes, including furins, stromelysin-1 (MMP-3), certain cathepsins, and plasmin, which cleave the inhibitory propeptide domain, thereby exposing the enzyme’s catalytic site. The activity of MMPs, including MMP-9, is tightly regulated by tissue inhibitors of metalloproteinases (TIMPs), particularly TIMP-1 to TIMP-3. Once activated, MMP-9 can amplify the inflammatory response by activating additional inflammatory mediators in the tear film, such as pro-interleukin-1β (pro-IL-1β), pro-tumor necrosis factor-alpha (pro-TNF-α), and substance P [81].

Elevated levels of MMP-9 have been documented in various subtypes of DED. While this protein is among the most extensively studied biomarkers in clinical research on DED, there remains ongoing debate regarding its diagnostic cut-off values, clinical relevance, and correlation with disease severity. These inconsistencies are likely attributable to the considerable variability in MMP-9 concentrations observed among DED patients. Notably, MMP-9 levels have shown a significant positive correlation with the OSDI and fluorescein staining scores and an inverse correlation with TBUT, Schirmer test results, and both conjunctival and corneal staining scores [82,83].

Tear film hyperosmolarity, a hallmark of DED, induces the release of inflammatory mediators and activates cellular stress pathways, promoting oxidative stress responses [38,74]. As individuals age, the ocular surface and tear film stability progressively deteriorate. A meta-analysis by Kitazawa et al. concluded that aging exerts a significant impact on the ocular surface microenvironment, contributing to the development of DED [15].

In the elderly, the infiltration of senescence-associated T cells, alongside other inflammatory cells, occurs in the ocular surface epithelium, lacrimal gland, and meibomian glands. These changes are accompanied by senescence-related markers, including the accumulation of 8-OHdG, lipofuscin-like inclusions, the increased expression of p53 and apoptosis-related genes, and decreased Ki67-positive cell populations. “Inflammaging,” a state of chronic, low-grade inflammation associated with aging [84], has emerged as a key molecular driver of DED in older adults [15,85]. Elevated levels of TNF-α have been reported in the lacrimal glands and tears of aged humans and animal models, contributing to chronic inflammation and tissue degeneration. In murine studies, TNF-α has been identified as a pivotal cytokine promoting age-related DED, with TNF-α modulation showing promise in attenuating DED phenotypes [71]. Oxidative stress also intensifies with age, leading to damage and inflammation of the lacrimal glands and ocular surface [73,74,86]. The accumulation of oxidative products such as 8-OHdG and lipofuscin-like inclusions, along with the buildup of senescent cells in ocular surface tissues, results in the secretion of pro-inflammatory factors known as the senescence-associated secretory phenotype (SASP), further promoting chronic inflammation [15].

Cathepsin S (CTSS), a lysosomal cysteine protease, has recently been identified as a novel therapeutic target for age-related DED. CTSS activity is significantly increased in aged tears and lacrimal glands, with CTSS−/− mice demonstrating resistance to corneal barrier disruption and goblet cell loss, highlighting the role of CTSS in age-associated ocular surface degeneration [86]. The therapeutic effects of a selective cathepsin S inhibitor, RO5459072, were investigated in a randomized, double-blind, placebo-controlled, parallel-group Phase IIA clinical trial [87] involving adult patients with moderate-to-severe primary SS. The primary endpoint was the proportion of patients achieving a ≥3-point reduction from the baseline in the EULAR SS Disease Activity Index (ESSDAI) score. Secondary outcomes included mean changes from the baseline at week 12 in the ESSDAI and the EULAR SS Patient Reported Index (ESSPRI), as well as measures of quality of life assessed via the Short Form-36 Health Survey (SF-36), including both physical and mental component scores. Additional secondary outcomes included unstimulated tear production and mechanically stimulated salivary flow. Although RO5459072 was found to be safe and well tolerated, the trial results did not demonstrate a clinically meaningful improvement in the ESSDAI score. Furthermore, no significant benefits were observed in any of the secondary clinical endpoints evaluated.

Proteomic analyses have revealed age-related alterations in tear composition, with changes in proteins involved in inflammation, immunity, and tissue repair [18,88]. S100A proteins have been implicated as potential molecular targets in ocular surface inflammatory diseases [89].

Overall, inflammation mediated by cytokines and chemokines, oxidative stress, and dysregulated proteolytic activity constitute the fundamental mechanisms underlying DED across all age groups, with distinct features emerging in the context of aging.

The identification of reliable biomarkers is essential for the diagnosis, monitoring, and development of targeted therapies for DED. Numerous biomarkers have been evaluated across ocular fluids and tissues, although many candidates still require immunological validation [12,64].

Tear hyperosmolarity and MMP-9 levels are elevated in DED and are currently utilized as diagnostic biomarkers. Osmolarity (TearLab) and MMP-9 (InflammaDry) are the only FDA-approved point-of-care (PoC) diagnostic tests for DED.

In addition, antimicrobial proteins such as lysozyme and lactoferrin are reduced in DED [90]. Lactoferrin concentrations can be measured using commercially available PoC devices, such as the ATD LTF™ Diagnostic Test Kit [91,92]. These biomarkers are typically used alongside clinical evaluations to enhance diagnostic accuracy.

Changes in the lipid composition of meibomian gland secretions, including alterations in glucosylceramides (GluCers) and monosialotetrahexosylganglioside 3 (GM3) [93], have been proposed as biomarkers, although validated diagnostic platforms are lacking.

Tear biomarkers common to DED and comorbid diseases, such as ocular allergy, include cytokines associated with Th1/Th17 (IL-1α, IL-1β, IL-17, TNFα, and IFNγ) and Th2 (IL-4, IL-5, and IL-13) pathways [94,95], as well as mucin alterations (MUC1, MUC2, MUC4, MUC5, and MUC16) [96,97,98,99]. Other proteins associated with inflammation and immune responses, including serum albumin (ALB), annexin A1 (ANXA1), neutrophil defensin 1 (DEFA1), lipocalin 2 (LCN2), serotransferrin (TF), mammaglobin-A (SCGB2A2), protein S100-A8 (S100A8), and protein S100-A9 (S100A9), are positively correlated with age in DED) [100].

In pediatric populations, tear hyperosmolarity has been reported as a biomarker in diabetic children [101], and inflammatory cytokines, along with meibomian gland morphology, are under investigation [65,102]. The concentrations of IL-17A, IL-6, and prostaglandin E2 (PGE2) increased significantly in tear samples after 3, 6, and 12 months of orthokeratology (OOK) wearing [103].

In young adults, elevated inflammatory markers have been observed in terminal video display users [104]. A recent study comparing ocular surface characteristics, tear protein profiles, and cytokine expression in young adults with and without EDE revealed that the upregulation of both pro-inflammatory and anti-inflammatory cytokines likely reflects an adaptive response aimed at maintaining ocular surface homeostasis [105].

**Figure 4:**
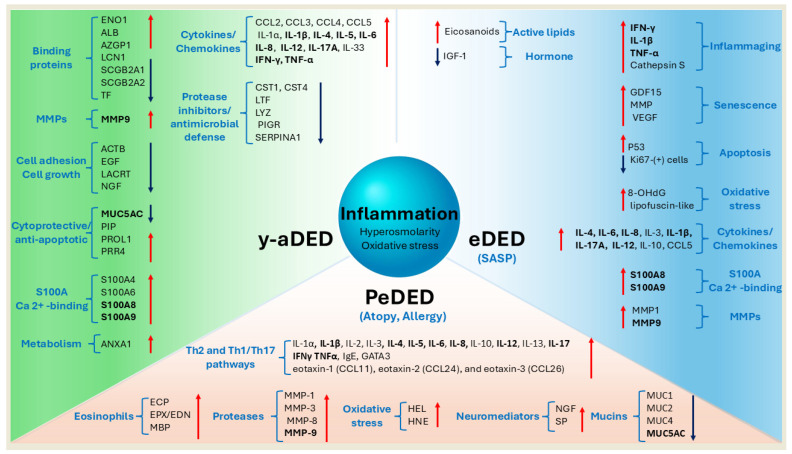
Molecular mechanisms and biomarkers involved in DED across age groups. Schematic representation of the principal molecular pathways implicated in the pathogenesis of DED in different age categories. Inflammation is a core mechanism across all age groups, often accompanied by hyperosmolarity and oxidative stress. Blue text indicates key biological processes, with specific molecules listed in brackets. Red arrows indicate proteins with increased expression; blue arrows denote decreased expression in DED. In PeDED, commonly linked to atopic and allergic conditions, the overexpression of inflammatory cytokines, neuromediators, proteases, and allergy-related molecules has been reported. In y-aDED, DED is marked by elevated levels of inflammatory cytokines and S100A family proteins, along with reduced expression of antimicrobial peptides and proteins involved in cell adhesion and growth. In eDED, activation of the senescence-associated secretory phenotype (SASP) leads to upregulation of molecules associated with chronic inflammation (inflammaging), oxidative stress, apoptosis, and specific bioactive lipids. Molecules consistently involved in all three age groups are highlighted in bold, suggesting early pathogenic mechanisms may persist and worsen with age if untreated.

In the elderly, beyond increased tear osmolarity and elevated inflammatory cytokines [90], additional age-related biomarkers have been identified. These include oxidative stress markers such as 8-hydroxy-2′-deoxyguanosine (8-OHdG), senescence-associated proteins, and distinct alterations in the tear proteome linked to age-related DED [15,86]. A meta-analysis of DED studies in human populations demonstrated that aging is associated with a decrease in tear volume, a reduction in TBUT, and an increase in tear osmolarity. Furthermore, analyses of tear fluid composition revealed significant increases in interleukins (IL-4, IL-6, IL-8, IL-3, IL-1β, IL-17A, IL-12, and IL-10), interferon-gamma (IFN-γ), TNF-α, chemokine (C–C motif) ligand 5 (CCL5, also known as RANTES), MMPs (MMP-1 and MMP-9), and eicosanoids. In contrast, levels of insulin-like growth factor 1 (IGF-1) in tears were found to decrease significantly, correlating with the presence and severity of DED [15,18,106,107,108]. Figure 4 presents proposed common and age-specific mechanisms and biomarkers associated with DED across different age groups.

Therapeutic strategies targeting senescence pathways, including senolytic approaches, represent a promising avenue for treating eDED. While limited, emerging evidence suggests that environmental stressors, such as digital device use, may promote premature cellular senescence on the ocular surface in younger populations.

## 10. Treatments

The management of DED aims to alleviate symptoms, restore tear film homeostasis, reduce inflammation, and prevent ocular surface damage [12,33,109]. Treatment strategies should be individualized based on the patient’s age, underlying etiology, comorbidities, and specific risk factors [10]. Therefore, it is crucial to consider age-related differences when selecting and tailoring therapies. A variety of prevention measures, pharmacological and non-pharmacological treatments, are proposed as an integral therapy for DED (Table 3).

### 10.1. Children

In pediatric populations, treatment often targets the underlying cause. This may include nutritional supplementation for VAD, the management of AC, and supportive care for SJS [19]. MGD is typically managed with warm compresses, lid hygiene, and, when necessary, topical antibiotics or corticosteroids [2,19]. Limiting digital screen time is an important modifiable risk factor.

General recommendations for the treatment of DED in children emphasize a multifaceted approach aimed at mitigating symptoms and addressing contributing factors. Behavior modification is essential, particularly encouraging frequent breaks during digital device use and promoting proper blinking habits to reduce tear film instability. The use of preservative-free artificial tears is recommended for symptom relief, minimizing the risk of ocular surface irritation associated with preservatives. Effective management of ocular and systemic allergies is crucial, as allergic inflammation can exacerbate DED symptoms. Additionally, maintaining gentle eyelid hygiene is beneficial, especially in cases of blepharitis or MGD, to support a healthy tear film. Environmental control measures, such as minimizing exposure to dry environments and tobacco smoke, are also important in reducing external stressors that contribute to ocular surface disease.

In addition to hygiene measures, it is essential to educate families to promptly recognize and address disease flares before inflammation becomes severe. Pharmacologic interventions range from artificial tears to calcineurin inhibitors such as tacrolimus, with corticosteroids used judiciously for short periods to minimize side effects. In AC, where signs of dry eye may overlap, accurate differential diagnosis and appropriate treatment are critical [121].

A recent Delphi consensus report [40] has outlined key strategies for the management of PeDED, emphasizing the importance of age-specific treatment algorithms. One central point of agreement among experts is that the primary indications for treatment are based on the presence of symptoms and ocular surface staining.

Importantly, treatment strategies must incorporate considerations of QoL, striving to balance clinical effectiveness with lifestyle impacts and avoiding unnecessary medicalization. For example, the use of eye drops, although often necessary, can sometimes adversely affect a child’s QoL. Artificial tears are frequently recommended in pediatric populations due to their ease of administration, favorable tolerability profile, and minimal disruption of tear clearance. Their main utility lies in symptom relief and mitigation of ocular surface damage, as indicated by staining.

In cases of PeDED associated with MGD, warming therapies are generally considered effective due to their ability to enhance the lipid layer of the tear film. However, consensus on their application in children aged 0–5 years was not achieved, likely reflecting concerns about treatment adherence in this age group. Macrolide antibiotics, both topical and oral, are recognized as valuable options in managing MGD-related PeDED. Topical macrolides are considered suitable across all pediatric age groups. In contrast, oral macrolides garnered consensus only for use in older children, with reservations for younger patients stemming from concerns about prolonged antibiotic use and the risk of resistance.

Topical corticosteroids play a limited role in PeDED treatment and require careful monitoring for potential adverse effects, especially in younger children, who may exhibit a heightened risk of steroid-induced complications. While corticosteroids are indispensable in severe cases such as blepharokeratoconjunctivitis, less aggressive approaches, such as artificial tears or topical macrolides, may be preferable in milder or ambiguous presentations. Overall, the findings underscore the necessity for individualized, age-appropriate management strategies that prioritize QoL. Further research is essential to validate these expert recommendations and to support the development of comprehensive, evidence-based guidelines tailored to pediatric populations.

### 10.2. Young Adults

Management in young adults typically includes artificial tears, preferably preservative-free for contact lens users, punctual plugs to reduce tear drainage, and topical anti-inflammatory agents such as corticosteroids (short-term) and immunomodulators like cyclosporine A [122,123,124]. The treatment of MGD with warm compresses, lid hygiene, and in-office procedures is often necessary [12]. Addressing modifiable lifestyle factors, such as excessive screen time, inadequate blinking, and contact lens hygiene, is crucial.

Emerging non-pharmacological therapies include intense pulsed light (IPL) therapy, which received FDA approval in 2021 for DED management. IPL reduces pro-inflammatory mediators such as IL-17A, IL-6, TNF-α, and MMP-9, thereby improving clinical outcomes in patients with MGD [125]. Clinically, IPL has been shown to diminish telangiectasias, eradicate *Demodex* mites, and liquefy meibum, contributing to its therapeutic benefits, and is considered a safe procedure. Although its precise mechanism of action remains unclear, the therapeutic potential of IPL was initially noted in patients treated for facial rosacea, particularly in the nasal and nasolabial areas, and has since been adopted in ophthalmic practice with favorable results.

In addition to IPL, several phytochemical agents, such as Manuka honey, aloe vera, and tea tree oil, have exhibited promising outcomes in preliminary studies. However, more robust, large-scale trials are needed. Finally, interventions that block blue light exposure have shown potential to improve visual acuity in patients with unstable tear films [31].

### 10.3. Elderly

Treatment in older adults must address multifactorial pathophysiology and comorbidities [10]. Preservative-free artificial tears are preferred to minimize ocular surface toxicity associated with chronic use of preserved formulations. Topical anti-inflammatory agents, including corticosteroids, cyclosporine A, lifitegrast, and tacrolimus in refractory cases, are commonly employed [112].

Due to the high prevalence of MGD, treatments such as warm compresses, eyelid massage, and in-office meibomian gland expression are often required [12]. Punctal plugs are useful in reducing tear drainage and improving ocular surface retention of natural and artificial tears.

A review and adjustment of systemic medications contributing to DED should be considered [56]. Systemic interventions such as oral vitamin D supplementation [126] and omega-3 fatty acids may support ocular surface health.

Emerging therapies, including TNF-α blockade, cathepsin inhibitors [71], lactobionic acid-based eye drops [32], and biologic tear substitutes (e.g., lactoferrin-containing formulations), are under investigation for age-related DED [127].

Inflammatory cytokines such as LPS, IL-1, TNF-α, and IFN-γ stimulate the expression and secretion of cathepsin S by macrophages, microglia, and epithelial cells [128].

Recent therapeutic innovations focus on developing comprehensive nanosystems to overcome ocular surface transmission barriers and disrupt the vicious cycle of DED pathogenesis. Additionally, regulating mitochondrial metabolites offers a promising anti-inflammatory strategy for ocular diseases [129].

## 11. Unmet Clinical Needs and Future Directions

Despite significant advances in the understanding and management of DED, several unmet clinical needs persist, particularly regarding age-related differences [12,130]. Early and age-specific diagnosis remains a critical gap. There is a pressing need for standardized and validated diagnostic criteria for pediatric populations, alongside a deeper understanding of the atypical clinical presentations of DED across different age groups. Additionally, more effective and targeted therapies are required, especially those addressing the specific pathophysiological mechanisms underlying DED in children, young adults, and the elderly, including therapeutic approaches aimed at senescence and inflammaging in older individuals.

A greater emphasis on the impact of DED on vision-related QoL is essential. Further research is needed to assess this impact on pediatric and young adult populations and to develop age-specific QoL assessment tools. In parallel, the management of DED in patients with coexisting systemic and ocular diseases, such as SS, glaucoma, and diabetes, requires dedicated guidelines tailored to different age groups.

Preventive strategies must be prioritized, particularly in children and young adults at high risk due to extensive digital device use and environmental exposures. Enhancing patient and healthcare professional education about DED across the lifespan is vital to promoting early detection and appropriate management.

Furthermore, given the increasing influence of modern lifestyle factors, including digital screen use and cosmetic practices, effective preventive and management strategies must be developed and implemented.

**Figure 5:**
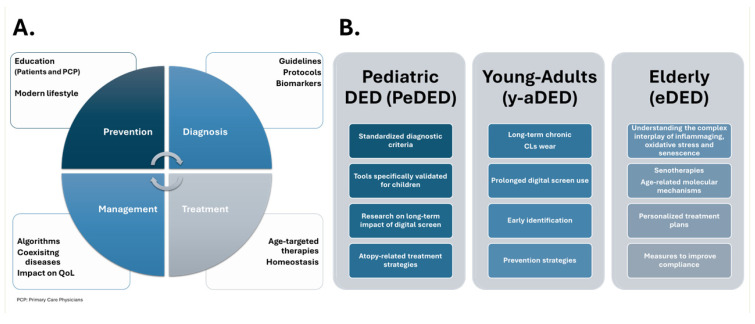
Unmet needs in the management of DED disease. (**A**) Proposed care model for DED: starting with prevention and education for patients and primary care providers, followed by standardized, biomarker-driven diagnosis; targeted treatment based on age, severity, and DED subtype; and comprehensive follow-up considering comorbidities and QoL. (**B**) Summary of key unmet clinical needs identified in each age-based DED category.

The identification and validation of age-specific biomarkers could significantly improve diagnosis, prognosis, and the evaluation of treatment responses across different patient populations. A proposed scheme for the management of DED is presented in Figure 5A.

In PeDED, standardized diagnostic tools specifically validated for children are urgently needed [40]. Further studies are required to elucidate the long-term impact of digital screen exposure on the developing ocular surface and to define the natural history of PeDED. Age-appropriate therapeutic strategies targeting the diverse etiologies of PeDED must also be developed.

In y-aDED, more research is needed to understand the long-term consequences of chronic contact lens use and prolonged digital screen exposure on ocular surface health. Early identification and preventive strategies are crucial, particularly for high-risk groups, such as VDT workers and contact lens wearers.

Regarding eDED, a better understanding of the interplay between inflammaging, oxidative stress, and cellular senescence in disease pathogenesis is critical. Moreover, the development of sensitive and specific biomarkers capable of predicting disease progression and therapeutic responses in the elderly is required. Novel therapies targeting key molecular pathways, such as TNF-α and cathepsin S, hold significant promises.

Improving symptom reporting in elderly patients, particularly those with reduced corneal sensitivity, is essential for accurate diagnosis and treatment. When designing personalized treatment strategies for older adults, special attention must be given to polypharmacy and the management of comorbid systemic conditions. Additionally, further research into the long-term efficacy and safety of anti-inflammatory therapies in the elderly population is warranted. Figure 5B presents the main unmet needs identified in each category classified according to age.

Emerging areas of interest include evaluating the role of DED in myopic children [131] and developing non-invasive methods for tear biomarker analysis. Tear collection remains a technological challenge, particularly for the elderly, due to reduced tear volume. Thus, innovative, non-invasive, reliable, and reproducible sampling techniques are urgently needed to facilitate biomarker discovery and clinical application.

Furthermore, an expert consensus on the proposed age-based classification of DED should be established in response to the evolving patient profile to define the selection of age-appropriate treatment strategies.

## 12. Discussion

This narrative review provides a comprehensive synthesis of the current knowledge regarding DED across the lifespan, highlighting significant age-related variations in prevalence, risk factors, clinical manifestations, and pathophysiological mechanisms. One of the most meaningful contributions of this work is the proposed age-based classification system, i.e., pediatric (PeDED), young adult (y-aDED), adult (aDED), and elderly (eDED), which underscores the need for tailored diagnostic and therapeutic approaches that align with the unique characteristics of each life stage.

Our findings reveal that while inflammation, tear film instability, and neurosensory abnormalities are common across all age groups, their relative contribution and clinical expression vary markedly with age. In pediatric populations, DED is closely linked to allergic and atopic conditions, while in young adults, lifestyle factors such as excessive screen use and contact lens wear dominate. In older adults, age-related structural changes, polypharmacy, and systemic comorbidities are primary contributors. Notably, emerging molecular evidence supports a central role for inflammaging, oxidative stress, and cellular senescence in eDED pathogenesis, with potential therapeutic implications.

Despite the growing body of research, several critical gaps remain. Evidence in children and young adults is still scarce, particularly regarding validated diagnostic tools and long-term outcomes. The absence of standardized, age-specific diagnostic criteria hampers early identification and intervention. Although a recently published expert consensus on PeDED emphasizes the need to standardize diagnostic and therapeutic practices to enhance the quality of life of affected children, the consensus statements still require rigorous validation before they can serve as the foundation for guidelines tailored to the specific needs of the pediatric population.

Although discovery technologies have uncovered numerous candidate tear-film biomarkers for dry eye disease, only a handful have advanced beyond initial identification owing to the absence of standardized sampling protocols, diagnostic cut-offs, and rigorous multicenter validation studies. Bridging this gap will require harmonized procedures, large-scale trials, multiplex assays, and computational modeling to establish clinically reliable biomarker panels that track disease severity and treatment response. Comparative studies evaluating treatment efficacy across age groups are also lacking, and current therapeutic guidelines are not adequately stratified by age.

The existing literature’s limitations include heterogeneity in diagnostic criteria, under-reporting of symptoms in certain populations (notably children and older adults with reduced corneal sensitivity), and insufficient inclusion of diverse geographic and socioeconomic contexts. These factors reduce the generalizability of findings and complicate cross-study comparisons.

This review itself has several limitations. Narrative reviews are inherently limited by their non-systematic methodology, which may introduce selection bias in the inclusion of the literature. Additionally, the absence of standardized criteria for study evaluation can affect the objectivity and reproducibility of the conclusions drawn. Another notable limitation of this narrative review on DED is the involvement of only two authors, each contributing expertise from distinct but complementary domains. While one author offers valuable insights into the clinical manifestations and real-life experiences of patients, the other focuses primarily on the molecular biology underlying the condition. This dual perspective, although enriching, may not fully capture the multifactorial and systemic nature of DED. Therefore, we invite a broad group of opinion leaders in this field to engage in a discussion of the issues raised. Nonetheless, this review’s strength lies in its integrative, age-focused approach, which brings attention to neglected patient subgroups and calls for consensus-driven strategies to guide future research and clinical practice.

## 13. Conclusions

DED demonstrates significant variation across the lifespan, with distinct patterns of prevalence, symptomatology, risk factors, pathophysiology, and molecular mechanisms in children, young adults, and the elderly. Although increasing attention is being given to DED in pediatric populations, its diagnosis and management require age-specific approaches. In children and young adults, the rising prevalence is largely attributable to atopy, allergy, and lifestyle factors such as prolonged digital device use and environmental exposures, while young adults are additionally at risk due to contact lens wear.

In contrast, advanced age remains a major risk factor for DED, driven by structural and functional changes in the lacrimal and meibomian glands, chronic low-grade inflammation, and cellular senescence. The elderly experience a heightened burden of disease resulting from the intricate interplay of inflammaging, senescence, and the effects of polypharmacy.

Recognizing and addressing these age-related differences is critical for the development of personalized and effective therapeutic strategies. Addressing the unmet clinical needs of DED across all age groups requires focused research efforts, age-adapted diagnostic tools and treatments, and care strategies that account for age-related differences in disease expression and therapeutic response. These efforts are essential to mitigating the burden of DED and enhancing ocular health and QoL throughout the lifespan. Also, given the rising prevalence across all age groups and the existence of modifiable risk factors, increasing societal awareness may help reduce the burden of this condition, which often requires prolonged and sometimes ineffective treatments that impact quality of life.

Despite the clear association between aging and DED, this relationship remains insufficiently explored, underscoring the urgent need for further multidisciplinary research. Moreover, although many candidate biomarkers have been identified, none have yet been conclusively validated for diagnosing or monitoring the disease. Thus, we recommend, firstly, that the clinical development of new agents and biomarkers tailored to specific age groups should be actively promoted. Secondly, a broader discussion should be initiated to facilitate the establishment of consensus-based working groups aimed at defining the management of DED across different age groups, including diagnosis, treatment with currently available agents, and related aspects such as biomarkers under development. Finally, multidisciplinary teams comprising pediatricians, geriatricians, primary care physicians, stakeholders, and patient associations should be engaged to raise awareness of the growing impact of this condition across the entire population.

## Figures and Tables

**Table 1:** Prevalence by region for children, youth, and the elderly.

Region/Country (Years)	Prevalence (%)	Reference
Global population	5–50	Stapleton et al. 2017 [5]
	5.5–65.4% (population-based studies)	
Global children (<18 y)	5.5–23.1	Stapleton et al. 2024 [14]
	23.7	Zou et al. 2025 [9]
Global ≥40 y	10–20	Britten-Jones et al. 2024 [11]
Global ≥50 y	5–30%	Stapleton et al. 2017 [5]
Global ≥60 y	9.2	Kitazawa et al. 2022 [15]
Global VDT users		
22–60 y	49.5	Courtin et al. 2016 [21]
20–58 y	26–70	Fjaervoll et al. 2022 [22]
Japan 20–60 y	10.1 (male)	Uchino et al. 2008 [23]
	21.5 (female)	
United States	8.1	McCann et al. 2022 [16]
>18 y	3.5	
>68 y	7.8	
Europe		
Spain	16.6 (WHS criteria)	Benítez-Del-Castillo and Burgos-Blasco 2025 [24]
	22.5 (BES criteria)	
18–29 y	5.7	
30–39 y	4.7	
40–49 y	9.8	
50–59 y	11.6	
60–69 y	17.1	
70–79 y	21.8	
≥80 y	26.4	
Norway		Tellefsen et al. 2021 [13]
20–39 y	24.1	
40–59 y	39.4	
≥60 y	36.5	
Rusia		Bikbov et al. 2022 [25]
≥84.5 y	35.8	
Asia		Cai et al. 2022 [26]
<20 y	11.9	
20–29 y	7.5	
30–39 y	7.7	
40–49 y	11.5	
50–59 y	13.3	
60–69 y	22.6	
≥70 y	28.9	
India		Donthineni et al. 2020 [19]
Total (<21 y)	0.4	
Infancy (<1 y)	0.05	
Toddlerhood (1–2 y)	0.01	
Early childhood (3–5 y)	0.02	
Middle childhood (6–11 y)	0.10	
Early adolescence (12–18 y)	0.15	
Late adolescence (19–21 y)	0.15	
China		Song et al. 2018 [27]
5–89 y	13.6 (by symptoms and signs)	
	31.4 (by symptoms)	
Central and South America		Chen et al. 2024 [28]
Brazil		Castro et al. 2018 [29]
≥18 y	13.0	
18–39 y	9.9	
40–60 y	13.2	
>60 y	21.1	
Mexico		Martinez et al. 2016 [30]
≥50 y	41.0	
46–55 y	36.0	
66–75 y	18.0	
76–85 y	38.0	

Abbreviations: BES: Beijing Eye Study; VDT: visual display terminal; WHS: Women’s Health Study; y: years.

**Table 2:** Coexisting diseases with DED.

Ocular/Systemic Disease	Children (<18 y)	Young Adults (18–40 y)	Adults (40–60 y)	Elderly (>60 y)
Atopic dermatitis				
Ocular allergy (AC, AKC)				
Stevens–Johnson syndrome				
Vitamin A deficiency				
Ocular allergy (VKC)				
Acne (early and late adolescence)				
Meibomian gland dysfunction				
Mental health disorders				
Altered sleep conditions				
Thyroid disorders				
Glaucoma				
Sjögren’s syndrome				
Diabetes mellitus				
Conjunctivochalasis				
Age-related macular degeneration				
Lupus erythematosus				
Rheumatoid arthritis				
Xerostomia				

Abbreviations: AC, allergic conjunctivitis; AKC, atopic keratoconjunctivitis; DED, dry eye disease; VKC, vernal keratoconjunctivitis. The blue color differences represent the increasing age of the population, from the light blue of children to the dark blue of the elderly.

**Table 3:** Therapeutical options for DED according to age.

Recommended and/or Available Treatments	Description	References
**Children (<18 years)**		
Lifestyle modifications	Reducing screen time, increasing outdoor activities, and mitigating environmental factors (exposure to air pollution)	Stapleton et al. 2024 [14]
Blinking exercises	Encouraged as part of lifestyle modifications	
Management of lid diseases (anterior blepharitis and MGD)	The role of MGD in PeDED is recognized, although the mechanisms may differ from adults	Villani et al. 2025 [40]
Warm compress therapy	Demonstrated efficacy in MGD in adults Compliance may be challenging, particularly in children (0–5 years), due to the difficulty of maintaining the required application time	Lazreg et al. 2024 [110]
Unpreserved lubricants or artificial tears	Commonly recommended due to their high tolerability and minimal impact on tear clearance Primarily used to relieve symptoms and improve ocular surface staining, particularly in mild DED	Buzzonetti et al. 2023 [111]
Management of ocular allergy	Identifying ocular allergy is crucial because it can exacerbate DED symptoms through tear film stability	Stapleton et al. 2024 [14] Villani 2025 et al. [40]
Topical macrolides	Azithromycin for MGD-related DED Preferable for managing chronic inflammation in certain cases like BKC	Villani et al. 2025 [40]
Oral macrolides	Azithromycin for MGD-related PeDED, with caution in younger children No consensus for children 0–5 years	
Topical steroids	Limited role in PeDED, with caution in younger children, due to safety concerns with prolonged use Use cautiously for BKC to maintain corneal clarity but limited in less clear-cut situations No consensus for children 6–15 years age	
Topical 0.2% HA Topical 0.2% HA with 0.1% arnica extract	Effective for TBUT, conjunctival redness, and symptoms measured via OSDI Topical 0.2% HA with 0.1% arnica extract showed a higher effect vs. HA alone	Buzzonetti et al. 2023 [111]
Vitamin A supplementation	Preventive for ocular morbidity due to DED in children and adolescents, particularly with VAD	Stapleton et al. 2024 [14]
Management of severe underlying conditions	SJS, the leading cause of severe ADDE and visual morbidity in younger children Ocular management of SJS is suggested as a preventive measure	Donthineni et al. 2020 [19]
Tear replacement, tear stimulation, or tear conservation approaches	No robust evidence Includes methods like punctal plugs	Stapleton et al. 2024 [14]
**Elderly (over 65 years)**		
**Conventional/Available Treatments**		
Artificial tears/lubricants	Commonly used for long-term therapy, often with glaucoma drugs Recommended as conventional, low-risk therapies in general DED management Their long-term use (“protective drops”) improve inflammation and provide symptom relief, particularly in elderly patients with conjunctivochalasis	Mohamed et al. 2022 [112] Ozek et al. 2020 [58]
Eye drops containing HA and LA	Greater and longer-lasting efficacy than HA alone in patients aged 56–80 years, likely because LA mimics the lactoferrin lacking in age-related DED	Gagliano et al. 2018 [32] Barabino 2022 [10]
Management of lid diseases	MGD	de Paiva 2017 [38]
**New Possible Treatments (based on new molecules/targets and inflammaging):**		
TNF modulation	TNF modulation during aging is suggested as a novel strategy for age-related DED; TNF is identified as a critical cytokine in age-related DED	Kelagere et al. 2023 [71]
CTSS modulation	CTSS modulation might be a novel target for age-related DED; increased CTSS activity levels have been found in aged tears and lacrimal gland lysates	Yu et al. 2022 [86]
**Challenges in Treatment Administration in the Elderly**		
Patient education on the correct technique for administering eye drops	Crucial for elderly patients who often require long-term eye drop use for conditions like DED and glaucoma Elderly subjects may not possess intrinsically good self-application techniques Clear, step-by-step instructions can significantly improve technique Public education programs on eyelid health and DED’s impact on the elderly’s lives are needed	Choy et al. 2019 [113] Abdelrahman et al. 2024 [114]
**Treatments Generally Applicable to Adult Age Groups (typically >18 y)**		
Patient education	Important for successful management To address that DED is often a chronic disease requiring long-term treatment	Mohamed et al. 2022 [112]
Environmental and lifestyle modifications	Decreasing exposure to high temperature, low humidity, air conditioning, smoke, pollution, and dust Reducing video display terminal use, taking periodic breaks, and performing blinking exercises	Lazreg et al. 2024 [110]
MGD treatment	Lid hygiene, warm compresses, and lid massage to relieve glandular obstruction and facilitate meibum outflow Level 2 studies on lid hygiene, including lid scrub ± massage or use of wipes/cleansing products	Narang et al. 2023 [115] Jones et al. 2017 [109]
Artificial tears/lubricants	Hypotonic solutions, those containing HA, vitamin A, taurine, dexpanthenol, or sodium carboxymethylcellulose to restore tear film homeostasis Preservative-free eyedrops are recommended	Teo et al. 2020 [116] Kathuria et al. 2021 [117] Lazreg et al. 2024 [110] Mohamed et al. 2022 [112]
Topical cyclosporine	Studies have evaluated its efficacy and safety, showing symptoms and signs benefits	Jones et al. 2017 [109] Lazreg et al. 2024 [110] Wei and Asbell 2014 [80]
Topical or oral azithromycin	Reported in blepharitis or severe blepharitis	Jones et al. 2017 [109]
Oral omega-3 EFA supplementation	Reduce signs and symptoms	
Newer therapies for EDE due to MGD (non-pharmacological)	Vectored thermal pulsation and IPL therapy	Narang et al. 2023 [115]
Management of underlying systemic diseases	Diabetes, thyroid diseases, systemic lupus erythematosus, rheumatoid arthritis, and SS can aggravate DED symptoms	Mohamed et al. 2022 [112] Rana et al. 2022 [118] Ziaragkali et al. 2018 [119]
Management of ocular comorbidities	Blepharitis, allergic conjunctivitis, and DED following LASIK or cataract surgery Treatment should be tailored by targeting disease mechanisms	Stapleton et al. 2024 [14] Mohamed et al. 2022 [112] Shi et al. 2023 [76] Mohamed et al. 2015 [120] Jones et al. 2017 [109]

Abbreviations: BKC, blepharokeratoconjunctivitis; CTSS, cathepsin S; EDE, evaporative dry eye; EFA, essential fatty acid; HA, hyaluronic acid; IPL, intense pulsed light; LA, lactobionic acid; MGD, meibomian gland dysfunction; SS, Sjögren’s syndrome; VAD, vitamin A deficiency.

## Data Availability

No new data were created or analyzed in this study. Data sharing is not applicable to this article.
